# Advancing Oral Health Equity Through School-Based Oral Health Programs: An Ecological Model and Review

**DOI:** 10.3389/fpubh.2019.00359

**Published:** 2019-11-26

**Authors:** Lynn Gargano, Margaret K. Mason, Mary E. Northridge

**Affiliations:** New York University (NYU) Langone Dental Medicine—Brooklyn, Hansjörg Wyss Department of Plastic Surgery, NYU School of Medicine, Brooklyn, NY, United States

**Keywords:** school-based oral health programs, oral health equity, common risk factor approach, ecological model, federally qualified health centers, oral disease prevention, dental services, interventions to reduce inequalities

## Abstract

In the United States and elsewhere, children are more likely to have poor oral health if they are homeless, poor, and/or members of racial/ethnic minority and immigrant populations who have suboptimal access to oral health care. As a result, poor oral health serves as the primary marker of social inequality. Here, the authors posit that school-based oral health programs that aim to purposefully address determinants of health care access, health and well-being, and skills-based health education across multiple levels of influence (individual/population, interpersonal, community, and societal/policy) may be more effective in achieving oral health equity than programs that solely target a single outcome (screening, education) or operate only on the individual level. An ecological model is derived from previously published multilevel frameworks and the World Health Organization (WHO) concept of a health-promoting school. The extant literature is then examined for examples of evaluated school-based oral health programs, their locations and outcomes(s)/determinant(s) of interest, the levels of influence they target, and their effectiveness and equity attributes. The authors view school-based oral health programs as vehicles for advancing oral health equity, since vulnerable children often lack access to any preventive or treatment services absent on-site care provision at schools. At the same time, they are incapable of achieving sustainable results without attention to multiple levels of influence. Policy solutions that improve the nutritional quality of children's diets in schools and neighborhoods and engage alternative providers at all levels of influence may be both effective and equitable.

## Introduction

Children who suffer from poor oral health are 12 times more likely to have restricted activity days than children with good oral health (1). Moreover, racial/ethnic minority children have been reported to experience 2 to 3 times poorer oral health compared to majority population children (2). Yet absent attention to the social determinants of health, school-based oral health education programs may not be accompanied by health gains (3), and when they do, they may actually exacerbate oral health inequalities (4). These sobering findings merit focused consideration of how schools may provide general and oral health care services on-site for vulnerable children, with the overall goal of advancing oral health equity and promoting social justice.

The particular focus of this review is on the underappreciated topic of oral health equity (5). Notwithstanding the provision of fluoride in its many forms (including through drinking water, fluoride varnish, and fluoridated toothpaste and mouthwash) (6) and improved detection and treatment modalities, dental caries remains a major public health challenge, affecting 60–90% of children worldwide (7). Indeed, while carious lesions are largely preventable—and even reversible—if detected in their early stages and when effective interventions are available, they remain the most common chronic health condition among school-aged children (8). If left untreated due to limited financial resources or lack of access to oral health care facilities, dental caries may cause pain and suffering, influence growth, development, and cognitive function, and lead to oral health problems in later life (9, 10). Since students with dental caries are more likely to miss school and perform poorly due to dental pain, improving their oral health is essential for enhancing their educational experience (11).

### An Ecological Model to Advance Oral Health Equity

**Figure 1:**
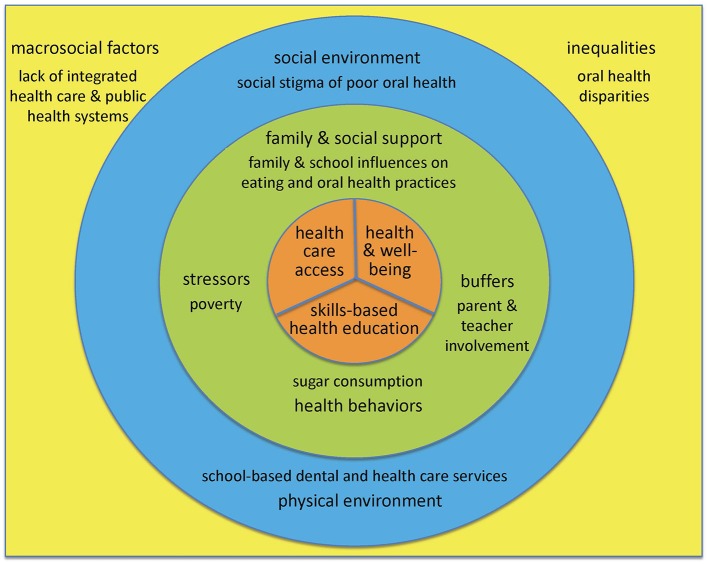
An Ecological Model to Advance Oral Health Equity. An ecological model of influences on health care access, skills-based health education, and health and well-being brought about by school-based oral health programs, adapted from previous work by the senior author (14–16), with key contributions from the World Health Organization concept of a health promoting school (17, 18).

Ecological models posit that factors at multiple levels influence disparities in access to and quality of services (12). Interventions that address factors at multiple levels may be more effective than those that target a single level (13). Figure 1 originated in a health promotion framework by the senior author that considers dynamic social processes through which social and environmental inequalities—and associated health disparities—are produced, reproduced, and potentially transformed (14, 15).

It was adapted from a multilevel model of influences on oral health and health disparities that highlighted family and social factors (16), also by the senior author, with important contributions from the World Health Organization (WHO) concept of a health promoting school toward realizing the goal of oral health equity (17, 18).

At the center of the ecological model are the 3 outcomes of interest potentially brought about by school-based oral health programs. First, school-based oral health programs may improve **health care access**, as schools are often the most efficient means of providing both preventive and treatment services for children (17, 18), especially for students from disadvantaged communities and families. Second, school-based oral health programs may improve general **health and well-being**, since oral health is fundamental to general health and has a well-documented impact on quality of life (18). Third, school-based oral health programs may improve **skills-based health education**, e.g., by enhancing understanding among students, teachers, and parents of factors that influence health, thus enabling them to make healthy choices and adopt healthy behaviors throughout their lives (17).

The next concentric circle in Figure 1 includes individual and interpersonal factors that affect these outcomes. Included therein are health behaviors, e.g., sugar consumption, as well as family and social support, e.g., influences on eating and oral health practices such as supervised toothbrushing with fluoride toothpaste, often constrained by socioeconomic conditions at home and school. For children, the attitudes, attributes, and knowledge of teachers and parents may be viewed as intermediary mechanisms through which societal and community influences affect their health and well-being (16). School-based oral health programs may achieve greater success if they take into account factors at the interpersonal level—including stressors on children and their families (homelessness) and potential buffers (parental involvement)—that may improve the outcomes of interest, rather than focus only individual level factors.

The last concentric circle in Figure 1 represents determinants of health- and education-related outcomes at the community level. Health promotion strategies that integrate elements of policy development and social and physical environmental factors may be yet more effective for oral disease prevention than isolated behavior-specific interventions (19, 20). In terms of the social environment, school-based oral health programs need to be cognizant of the social stigma associated with poor oral health, and address student and parental concerns regarding appearance as well as health (18). In terms of the physical environment, the available resources in given schools, notably space, may determine in part whether school-based oral health programs operate within a van or mobile bus, utilize portable equipment, or maintain fixed sites (21, 22).

Finally, the square in Figure 1 that contains the full model represents the societal level influences that school-based oral health programs must necessarily contend with and potentially help transform. These include macrosocial factors such as the lack of integrated medical and dental health care systems and the historical neglect of oral health within public health policies and programs. Instead, stakeholders at all levels would do well to tackle the underlying social determinants of oral and general health through the adoption of the common risk factor approach, emphasizing diet, hygiene, tobacco and alcohol use, stress, and trauma (23). Finally, inequalities in the distribution of wealth, education, and power as expressed through enduring health disparities reflect the universal social gradient and underscore the influence of economic and political determinants of oral and general health (24).

## Selected Models of Evaluated School-based Oral Health Programs

Models of evaluated school-based oral health programs were selected through a directed online search of the open access PubMed repository, using Figure 1 as the guide to identify search terms. It is important to emphasize that absent a rigorous evaluation, programs were not included in this analytic review. The literature search was not strictly linear as with a systematic review. Rather, apt examples of programs targeting single or multiple outcomes, and single or multiple levels of influence, were purposefully sought to illustrate the utility of Figure 1. It was also deemed critical to the authors to include programs at various scales (local, regional, national) and geographies. The findings were organized first by outcome(s) and then by level(s) of influence targeted by the programs, and presented by beginning at the center of Figure 1 and moving outward to encompass increasingly higher levels of influence (Table 1).

**Table 1:** Selected models of school-based oral health programs, along with their locations, outcome(s)/determinant(s) of interest, level(s) of influence, and effectiveness and equity attributes.

**References**	**Location**	**Outcome(s)/Determinant(s)**	**Level(s) of influence**	**Effectiveness attributes**	**Equity attributes**
**Screening**					
Detty (25)	Ohio, USA	Screening to determine measures of health and well-being [oral health and height/weight to determine body mass index (BMI)]	Individual/population	Screening in isolation only provides data for monitoring the burden of disease, without improving the outcomes of interest	Lower participation rates were found with high student mobility and large school size
**Skills-based health education**					
Blake et al. (26)	East Midlands, UK	Skills-based health education (single interactive evidence-based educational session)	Individual, interpersonal	Short-term improvements in children's knowledge of oral health and use of dental floss, but not in toothbrushing or dietary behaviors	100% participation rate was achieved, ensuring that all children received the intervention
Jaime et al. (27)	Monte Sião, Brazil	Skills-based health education [(1) the school dentist initially gave a lecture to the children's parents; (2) the school dentist then gave a short course to the school staff; and (3) the trained teachers subsequently delivered a 6-module course to the students]	Individual, interpersonal	After 3 years, caries incidence was not decreased, but slight improvements in health knowledge were found (i.e., knowledge of dental caries and use of dental floss every day)	Monte Sião has a Human Development Index of 0.811 (slightly higher than the Brazilian average of 0.792) and fluoridated water
Esan et al. (28)	Ile-Ife, Nigeria	Skills-based health education (school-based oral health education curriculum for primary school children, complemented by a community-based educational program targeted to 3 groups: pregnant women, parents, and teachers)	Individual, interpersonal, community	After 4 years, the results were mixed: use of fluoridated toothpaste and toothbrushing twice daily improved, but not consumption of sugary snacks or daily flossing	The inability of control participants to purchase toothbrushes and other commodities for preventive oral self-care may have biased the findings
Saied-Moallemi et al. (29)	Tehran, Iran	Skills-based health education (the intervention consisted of illustrative puzzles as learning tools for the children and an oral health educational leaflet and brushing diary for the parents)	Individual, interpersonal	During the 3-month school-based intervention, 60% of the children in the intervention group and 32% of the children in the control group achieved a healthy gingiva	Girls experienced fewer barriers to dental care after the program and were more likely to achieve a healthy gingiva than boys
**School influences on oral health practices**					
Lai et al. (30)	Taipei area, Taiwan	School influences on oral health practices (meticulous instructions on finger flossing the Bass method of brushing of children originally aged 10-11 years, guided and monitored by dentists and school nurses; intensive oral health education by school nurses and dental concepts by dentists)	Individual, interpersonal	After 10 years of follow-up, positive effects were found on the dental knowledge, oral hygiene habits, plaque scores, periodontal status, caries experience, and preventive dental visits of children in the intervention group	Parents' educational level did not differ between the intervention and control groups
**School-based dental and health care services**					
Larsen et al. (31)	New York, NY, USA	School-based dental and health care services (largely diagnostic, preventive, and restorative, with small percentages of endodontics, periodontics, surgery, and adjunctive services delivered by dentists and dental hygienists)	Individual, interpersonal, community	School-based clinics are more productive, efficient, and cost-effective than community-based clinics in providing dental care to underserved children, and perform more preventive services	Transportation issues, parent availability, and missed appointments are greatly reduced in school-based health clinics
Carpino et al. (32)	Kansas City, MO, USA	School-based dental and health care services (examinations, oral health instructions, prophylaxis, fluoride applications, restorations, sealants, emergency visits, extractions, and endodontic treatment delivered by dental and dental hygiene students)	Individual, interpersonal, community	As the encounter intensity with a dental or dental hygiene student increased, so too did the oral health of children (decrease in decay, increase in restorations, and reduction in referral urgency)	Only 27.9% of eligible students participated in the program, the vast majority of whom qualified for the free or reduced lunch program
Amalia et al. (33)	Province of Yogyakarta, island of Java, Indonesia	School-based dental and health care services (oral health screening followed by oral health education in classrooms at least twice a year; classroom tooth brushing; children identified with caries receive full treatment upon referral; and all teachers are trained in oral health matters at least once a year)	Individual, interpersonal, community	Children participating in a poorly performing program had a greater likelihood of experiencing an oral impact on their quality of life, but difficulty with eating was higher (42.4%) for good programs than for poor programs (38.6%)	Living in a rural area and being a girl were significantly associated with a greater risk of having a lower quality of life
Culler et al. (34)	Chelsea, MA, USA	School-based dental and health care services [annual oral health education, dental screening and referral, and application of dental sealants and fluoride varnish; preventive and restorative treatment (exams, cleanings, and fillings) provided at a centrally located, full-service, school-based dental clinic open year-round]	Individual/population, interpersonal, community	A greater percentage of Chelsea students had untreated decay and severe treatment need than students statewide, but fewer Chelsea third graders had severe treatment need and more had dental sealants	There was no significant difference in the percentage of Chelsea students having severe treatment need or dental sealants by income level
**Multilevel interventions**					
Muirhead and Lawrence (35)	York Region, Ontario, Canada	Multi-level intervention (the Healthy Schools recognition program is a voluntary program managed by the 72 Ontario school boards; existing or proposed health-related activities included healthy eating, physical activity, bullying prevention, personal safety and injury prevention, substance use and abuse, healthy growth and development and mental health activities)	Individual/population, interpersonal, community, societal/policy	School participation/neighborhood socioeconomic factor interactions were present: a lower percentage of children in low-income “Healthy Schools” had preventive and urgent dental treatment needs and 2 or more decayed teeth than in low-income non-participating schools	Schools situated in poorer neighborhoods may benefit more from health promotion activities than schools situated in more affluent neighborhoods
Edasseri et al. (19)	Greater Montreal, Québec, Canada	Multi-level intervention (the QUALITY cohort recruited 630 white children aged 8-10 years at baseline from schools located within 75 km of 3 urban centers in the province of Québec; trained dentists performed the clinical oral health examination in a dental office during the hospital visit)	Individual/population, interpersonal, community, societal/policy	Children attending schools with strong healthy eating programs had lower 2-year incidence of dental caries than schools with weak programs	Greatest protection against dental caries was found with both strong healthy eating environments inside the schools and favorable food environments around the schools
Ariga et al. (36)	Kuwait	Multi-level intervention (School Oral Health Program, Kuwait, delivers oral health education, prevention, and treatment to 280,000 school-aged children; delivery of care is through a system of center- and school-based clinics and preventive mobile teams that deliver preventive services to children in schools without permanent dental clinics)	Individual/population, interpersonal, community, societal/policy	Effective primary prevention of dental caries is emphasized, resulting in a considerable reduction in treatment needs, which is evident from the reduced number of composite restorations performed under this program during the last 6 years	Prevention coverage (biannual application of fluoride varnish and the placement of pit and fissure sealants on newly erupted permanent molars and premolars) is close to 80%.
Wolff et al. (37)	Grenada	Multi-level intervention (toothbrushing in the classrooms, fluoride varnish applications, and placement of sealants; use of fluoride varnish in the hands of lay professionals provides a simple and safe caries-preventive therapy that can be utilized as a public health intervention through different providers when governmental organizations recognize that the benefits can far outweigh minimal risks)	Individual/population, interpersonal, community, societal/policy	The results of the pre- and post-caries surveys indicated a reduction in caries incidence, yet a high volume, less labor-intensive sealant technique could not be effectively achieved when sealant retention was utilized as an outcome measure	Increased awareness of good oral health among children, teachers, parents, and local health care providers, and trained teachers and local providers to deliver the intervention toward improving its sustainability

For each factor in Figure 1, evaluated school-based oral health programs with the designated outcomes and determinants of interest at the individual/population, interpersonal, community, and societal/policy levels of influence were critiqued in terms of both their effectiveness and equity attributes.

### Screening to Determine Health and Well-Being

At a minimum, school-based oral health programs might screen a population of children to determine their health and well-being. For example, the Ohio Department of Health conducted a statewide oral health and body mass index (BMI) screening survey among third grade children during the 2009–2010 school year (Table 1) (25). Of interest is that oral health and BMI were screened for together (25). As per the common risk factor approach (23), both dental caries and obesity may be prevented by focusing on the health behavior of lowering sugar consumption. Nonetheless, screening in isolation only provides data for monitoring the burden of disease, without improving the outcomes of interest or promoting oral health equity. Notably, schools with high student mobility and large school size had lower participation rates in this state-wide screening program (25).

### Skills-Based Health Education

Certain school-based interventions focus on skills-based education, largely at the individual and interpersonal levels, as exemplified by the 4 programs presented in Table 1 (26–29). A multi-site cohort study conducted with children aged 10–11 years who attended 1 of 3 primary schools in the East Midlands, UK used a single interactive, evidence-based educational session lasting ~60 min delivered by a dental care professional (26). A 3-year retrospective controlled clinical trial assessed the effect of a school-based oral health program on caries incidence of children aged 5–7 years from 2 public schools in Monte Sião, Brazil (27). A structured school-based oral health education program was implemented over 4 years in 6 primary schools in Ile-Ife, Nigeria (28), where a community-based education program complemented the school-based program by including pregnant women who attended antenatal clinics, providing general public education for parents to help dispel myths and misconceptions, and training teachers on the use of the curriculum (28). Finally, a school-based program using illustrative puzzles with a parental component comprised of a leaflet and brushing diary was designed for children aged 9 years in public primary schools in Tehran, Iran, where schools were randomly assigned to the intervention (4 boys' and 4 girls' schools) or control groups (2 boys' and 2 girls' schools) (29).

Overall, the 4 school-based health education programs reviewed were at most modestly effective in improving oral health-related knowledge and oral hygiene behaviors (26–29), mixed for health and well-being [no decrease in caries incidence achieved (27), but improvements in healthy gingiva found (29)], and health care access [fewer barriers to dental care (29)]. In terms of equity attributes, by conducting the programs in schools, most students received any associated benefits, except when the control participants or their families lacked the resources to purchase toothbrushes and other commodities for preventive oral self-care, or when boys and girls attended separate schools.

### School Influences on Oral Health Practices

School influences on oral health practices are considered to be part of the interpersonal level in Figure 1 (a form of family and social support). From 1993–2005, the Taiwanese government.

Launched an intensive oral hygiene education program for schoolchildren originally aged 10–11 years (30) (Table 1). Approximately 10 children in each of 12 schools for a total of 120 children were selected to receive the intervention, which consisted of meticulous instructions on finger flossing and the Bass method of brushing delivered by dentists, a semester-long oral health education program delivered by school nurses, and dental concepts delivered by dentists. The age, gender, and school grade matched control group children carried out their oral hygiene procedures in their own ways, but still supervised by school nurses. In terms of effectiveness, after an average of 10 years of follow-up (range = 4–16 years), positive effects on dental knowledge, oral hygiene habits, plaque scores, periodontal status, caries experience, and preventive dental visits were found in the intervention group (30). In terms of equity, no statistically significant difference in the parents' educational levels were found between the intervention and control groups.

### School-based Dental and Health Care Services

Various models exist for providing preventive and restorative dental services for students in schools, part of the community level in Figure 1 that includes the physical environment (14, 15). Evaluations of their effectiveness have generally shown improvements in oral health among the children served, but limited coverage of student populations and services provided, as per the 4 evaluations reviewed in Table 1 (31–34). In the first evaluation, the productivity, types of services, and cost-effectiveness of 4 dental clinics in schools were compared to 3 dental clinics in community-based health centers in New York City over a 12-month period (31). In the second evaluation, a repeated-measures design was used to longitudinally examine secondary data from participants at a dental clinic situated within a school-based health center and operated by the University of Missouri–Kansas City School of Dentistry (32). In the third evaluation, a cross-sectional survey was administered to students aged 12 years in 4 programs chosen to represent good and poor performance in urban and rural areas in Yogyakarta, Indonesia (33). The final evaluation was conducted of the Boston University/Chelsea Partnership Dental Program operating in Chelsea, MA, with a low-income and largely racial/ethnic minority population, by comparing participants' oral health to a Massachusetts state-wide oral health assessment (34).

In terms of effectiveness, schools act as an accessible location to provide preventive and responsive oral health care services to students (31–34). With regard to equity, however, school-based oral health programs on their own cannot overcome the challenges of the social determinants of oral health, including poverty (31–34), living in a rural location (33), or gender bias (33).

### Multilevel Interventions

Only 4 studies found in the published literature examined multilevel interventions on children's oral health, and thus were better able to assess impacts on oral health equity (19, 35–37) (Table 1). An ecological study was conducted in the York Region of Ontario, Canada that compared school-level oral health outcomes in elementary schools participating in Ontario's Healthy Schools recognition program and non-participating schools, and examined the effect of neighborhood socioeconomic status on oral health outcomes (35). A second multilevel Canadian study examined oral health promoting school environments and dental caries in Québec children using data from a cohort study that recruited white children at risk of obesity and their families from Greater Montreal schools (19). Third, the School Oral Health Program, Kuwait, delivers skills-based educational activities to 280,000 public schoolchildren (including supervised toothbrushing), parents, and expectant mothers, along with prevention and treatment services delivered through a system of center- and school-based clinics and preventive mobile teams (36). Finally, the Smile Granada program was implemented nationally from 2010 to 2013 for children aged 6–8 and 14–15 years, consisting of a daily 2-min toothbrushing routine using fluoridated toothpaste in every classroom, fluoride varnish applications 3–4 times per year, and glass ionomer sealants applied to the first permanent molars of children aged 6–8 years (37).

Thus, at both the regional (19, 35) and national levels (36, 37), school-based oral health programs have proven to be effective and equitable. Vulnerable children may lack access to any oral health preventive and treatment services without on-site care provision at schools. Still, they are incapable of achieving sustainable results without attention to multiple levels of influence.

## Discussion

The implications of this review for the oral health and well-being of vulnerable children are potentially transformative, since all levels of determinants in Figure 1 affect or are affected by school-based oral health programs. The included papers were selected to encompass several countries at different levels of socioeconomic development, and thus at different stages of demographic, epidemiologic, and nutritional transitions, in order to illustrate the utility of the ecological model in diverse contexts. Clearly, there are limitations to children learning about better oral hygiene and how to eat healthy, while vending machines and the menus at school cafeterias continue to promote unhealthy foods. Further, the harsh socioeconomic conditions of students at home may prevent them from achieving and maintaining proper oral health and nutritional eating behaviors. It is thus not surprising that homeless children suffer from higher obesity and dental caries rates than the US national childhood averages (38).

### Policy Solutions to Improve Nutrition and Oral Health

Foremost among potential policy solutions are efforts focused on improving the nutritional quality of children's diets in schools and their surrounding neighborhoods (19, 35). Indeed, healthy eating environments may have effects beyond obesity reduction, benefitting children's oral health (19, 35). In addition to funding school-based oral health programs for caries reduction, resources ought to be directed to strategies that embrace the common risk factor and participatory approaches, with a special emphasis on disadvantaged schools and neighborhoods (19).

Another proposed policy solution is a universal pre-Kindergarten to 8th grade caries prevention program that is school-based and delivered twice a year (39). The bundle of preventive services might include: screening; silver diamine fluoride treatment of all caries, pits, and fissures; fluoride varnish; oral hygiene instruction; and provision of a toothbrush and fluoride toothpaste (39).

### Use Alternative Providers to Provide Oral Health Education, Promotion, and Care

While dental hygienists (40, 41), including expanded care dental hygienists (42, 43), and mid-level providers such as dental therapists (44, 45) have met with successes and challenges in delivering school-based dental care, the universe of potential alternative providers is larger still. Teachers, school health nurses, and community health educators/workers (37, 46–48) are capable of reaching immigrant and other marginalized communities in need of improved health care access, health and well-being, and skills-based health education. For school-based oral health programs to be sustainable, it is important to create health teams with the active engagement of leaders at all levels, including health officials at the local, state, and national levels (37, 49).

### Expand the Capacity of Federally Qualified Health Centers (FQHCs) to Provide Oral Health Care Services

FQHCs are community-based health centers that receive funds from the Health Resources and Services Administration (HRSA) Health Center Program to provide primary care services in underserved areas, regardless of the ability of individuals to pay for services, insurance coverage, or citizenship status (50, 51). They may also potentially fill an important role in increasing access to oral health care for vulnerable and underserved populations, including impoverished children and their families. For example, FQHCs are required to provide certain services—including preventive, but not comprehensive, dental services—either in the clinic or by referral (52). The Institute of Medicine and the National Research Council recommend that HRSA expand the capacity of FQHCs to deliver essential oral health services by: supporting the use of a variety of oral health care professionals; enhancing financial incentives to attract and retain more oral health care professionals; providing guidance to implement best practices in management, operation, and efficiency; and assisting FQHCs in all states to operate programs outside of their physical facilities and leverage new systems to improve the oral health of the populations they serve (52). Notably, school-based oral health programs may either be operated by FQHCs (53) or partner with them for referral of student participants and their family members, so that everyone eventually secures a dental home.

### Research Priorities

Finally, it will be important to critically evaluate what is working well and what is not, and rely on evidence-based strategies to achieve oral health equity through school-based oral health programs. Recommendations include: (1) engage community stakeholders in intervention development and implementation; (2) train teachers, parents, and community activists to counter myths regarding the effectiveness of fluoride in preventing and reversing carious lesions; (3) collect and report economic and cost-effectiveness data, which is especially important to policy makers; and (4) strengthen the scientific approaches used, including implementation science and community-based participatory research (47, 48).

## Limitations

Figure 1 is an incomplete accounting of the determinants at multiple levels that move individuals/populations toward or away from oral health equity. Likewise, the model programs cited are illustrative of evaluated school-based oral health programs that target specific or multiple levels of influence and were available in PubMed at the time this article was written. Recent and other apt evaluated school-based oral health program reports have been omitted from this review, as the intent is to be illustrative of programs targeting single or multiple outcomes, and single or multiple levels of influence as per Figure 1, rather than comprehensive.

## Conclusion

School-based oral health programs that target multiple levels of determinants of health care access, health and well-being, and skills-based health education may not only be more effective than those that target only one level, but they may also be more equitable in terms of health and education outcomes and more just for children throughout the social gradient. Policy solutions that improve the nutritional quality of children's diets in schools and their surrounding neighborhoods and engage alternative providers at all levels of influence are priorities toward realizing sustainable improvements in the health and well-being of disadvantaged children and their families.

## Author Contributions

LG, MM, and MN each contributed to the conceptualization, research, critical analysis, and writing of the article, and take public responsibility for its content.

### Conflict of Interest

The authors declare that the research was conducted in the absence of any commercial or financial relationships that could be construed as a potential conflict of interest.
